# Editorial for the Special Issue “Microbial Solutions for Sustainable Resource Recovery and Environmental Remediation”

**DOI:** 10.3390/microorganisms14051008

**Published:** 2026-04-30

**Authors:** Chunqiao Xiao

**Affiliations:** School of Environmental Ecology and Biological Engineering, Wuhan Institute of Technology, Wuhan 430205, China; chunqiao@wit.edu.cn

In response to the triple crises of global resource depletion, escalating waste generation, and environmental pollution, microorganisms are increasingly transitioning from basic research to engineering applications, driven by their metabolic diversity, robust environmental adaptability, and modifiable engineering characteristics. This Special Issue, titled “Microbial Solutions for Sustainable Resource Recovery and Environmental Remediation”, aims to compile cutting-edge research findings and demonstrate how microorganisms can facilitate circular economy principles and promote green, sustainable development across domains such as metal recycling, pollutant degradation, waste resource utilization, bioenergy production, and nutrient cycling.

With the rapid advancement of global industrialization and urbanization, microorganisms are becoming the core force in developing sustainable bioprocesses and circular economy systems. This Special Issue comprises a total of eight high-quality papers, spanning multiple directions including bioleaching, microbial degradation, anaerobic digestion, microbial-induced carbonate precipitation (MICP), metagenomic analysis, mutagenesis-based strain improvement, and bibliometrics. The aim is to showcase the latest advancements of microorganisms in metal recycling, pollutant degradation, waste resource utilization, and innovations in systems biology, while exploring their practical applications in environmental sustainability.

In the field of bioleaching and metal recovery, this issue presents several inspiring studies. For example, a study on bacteria in the polluted sediments of Šibenik Bay has revealed how these microorganisms respond to toxic metal stress through multi-metal resistance mechanisms, providing new germplasm resources for the development of in situ remediation strategies for heavy metal-contaminated water bodies (Contribution 3). Additionally, the metagenomic analysis of dynamic changes in antibiotic resistance genes and heavy metal resistance genes during the composting process of phosphorus tailings (Contribution 6) deepened our understanding of lateral gene transfer risks during waste co-treatment and provided a scientific basis for optimizing composting processes to reduce the transmission risk of environmental resistance genes.

In the field of microbial degradation and waste treatment, this Special Issue particularly focuses on the degradation bottlenecks associated with low temperatures and complex substrates. A *Paenarthrobacter nitroguajacolicus* mutant, obtained via ARTP (atmospheric and room-temperature plasma) mutagenesis technology, was investigated to demonstrate how it enhances lignocellulose degradation at low temperatures. Comprehensive physicochemical property analysis, comparative genomics, and transcriptional expression profiling were conducted to verify this mechanism, providing an efficient strain for the rapid decomposition of agricultural straw in cold regions (Contribution 2). In response to widespread concern over microplastic pollution, a review systematically evaluates the impact of microplastics on the anaerobic digestion process, and their potential mechanisms of action, and highlights the threat posed by microplastic–microorganism interactions to biogas yield and system stability (Contribution 7).

Microbial solutions for resource recycling and the circular economy also present significant highlights. For example, *Bacillus velezensis* GY1, isolated from pig manure digestate, exhibits efficient ammonium nitrogen metabolism capacity and is capable of co-producing γ-polyglutamic acid (γ-PGA), thereby opening a new avenue for nitrogen recovery and high-value utilization in livestock and poultry-breeding wastewater (Contribution 1). In the realm of bioenergy and nutrient cycling, a bibliometric and visual review (Contribution 8) on three obligate organohalide-respiring bacterial genera systematically identifies research hotspots and evolutionary trends of these microorganisms in the fields of organic pollutant bioremediation, providing a clear academic roadmap for subsequent researchers.

Significant advancements have also been made in MICP technology for environmental remediation during this period. On one hand, Proteobacteria from limestone mines were screened and characterized, demonstrating their adaptability and potential for in situ mineralization remediation (Contribution 4). On the other hand, an MICP process driven by urea hydrolysis in thermophilic facultative anaerobic bacteria has been successfully developed under high-temperature anaerobic conditions, broadening the application scenarios of MICP in high-temperature industrial environments or geothermal settings (Contribution 5).

At the level of interdisciplinary innovation and synthetic biology applications, although this Special Issue does not include complete original research papers, many contributions have widely adopted modern methods such as metagenomics (Contribution 6), comparative genomics and transcriptional regulation analysis (Contribution 2), and bibliometrics (Contribution 8). This fully demonstrates the deep integration of environmental microbiology with systems biology and data science. These advancements indicate that designing microbial communities and reconstructing metabolic networks through synthetic biology will provide more powerful tools for achieving precise, efficient, and controllable environmental bioremediation and resource recovery.

The eight contributions in this Special Issue collectively highlight several cross-cutting themes: the conversion of waste into high-value products (γ-PGA production and low-temperature lignocellulose degradation), the co-selection risks associated with heavy metals and resistance genes, the expansion of MICP application boundaries under diverse conditions, and the integration of emerging contaminants and domain knowledge graphs. Rather than merely listing individual studies, the issue synthesizes key advances in sustainable resource recovery and environmental remediation.

Looking forward, several challenges remain. These include the need for the more robust engineering of microbial consortia for field-scale applications, a better understanding of the long-term ecological impacts of introduced microorganisms, and the integration of multi-omics and modeling approaches to predict system behavior under dynamic environmental conditions. Addressing these gaps will be critical for translating microbial solutions into practical, scalable, and economically viable technologies.

